# A Clinicopathological Analysis of Membranous Nephropathy and Its Correlation With the Immunohistochemical Expression of Phospholipase A2 Receptor (PLA2R) in Renal Biopsies in a Tertiary Care Center

**DOI:** 10.7759/cureus.70960

**Published:** 2024-10-06

**Authors:** Hemapriya K, Subalakshmi Balasubramanian, Barathi Gunabooshanam, Ponneyinchelvi AS, Pavithra V, Ram P Elumalai

**Affiliations:** 1 Pathology, Sri Ramachandra Institute of Higher Education and Research, Chennai, IND; 2 Pathology, Coimbatore Kidney Centre, Coimbatore, IND; 3 Nephrology, Sri Ramachandra Institute of Higher Education and Research, Chennai, IND

**Keywords:** membranous nephropathy, m type phospholipase a2 receptor (pla2r), pla2r-associated primary membranous nephropathy, primary membranous nephropathy, secondary membranous nephropathy

## Abstract

Background

Membranous nephropathy (MN) is the second most common adult-onset nephrotic syndrome worldwide. Traditionally, these were classified as primary and secondary, with primary causes showing higher positivity for various antigens, including M-type phospholipase A2 receptor (PLA2R), a key antigen located in the podocyte against which antibodies are directed.

Aim

The aim of this study is to analyze the cases diagnosed as MN with clinicopathological parameters and PLA2R positivity by immunohistochemistry in renal biopsies.

Methods

A retrospective observational study of 65 cases of MN diagnosed in renal biopsies by light microscopy and confirmed by ancillary studies from the Department of Pathology, Sri Ramachandra Medical College and Research Institute over a period of three years (2021-2023). The demographic profile and patient details were obtained from the hospital information system and archival case files. The description of categorical variables was expressed as frequency and percentage. The Chi-squared test and Fisher's exact test were employed to compare the distribution of qualitative variables between the groups.

Results

This case study includes 65 membranous nephropathy cases, of which 47.7% were diagnosed as primary MN and 52.3% as secondary MN. Of these, serum antinuclear antibody (ANA) positivity was seen in 80.6% of cases of primary MN and 58.8% of cases of secondary MN. Elevated serum C3/C4 levels were noted in 51.6% of primary MN and 47.1% of secondary MN (Ref. C3 = 90-180mg/dl; C4 = 10-40mg/dl). Immunofluorescence for IgG showed a nonspecific association between primary and secondary MN. Immunohistochemistry (IHC) for PLA2R showed positivity in 72.3% of primary MN cases and 27.7% of secondary MN cases. The Chi-squared test and Fisher's exact test showed statistical significance for these parameters.

Conclusion

This study signifies that primary MN is more frequently associated with positive immunohistochemical expression of PLA2R. These findings help in characterizing these cases as antigen-specific and have helped in the ongoing validation of PLA2R IHC as a diagnostic marker, which aids in monitoring the disease progression, remission, and recurrence. Despite the availability of various modalities for estimating the levels of anti-PLA2R, diagnostic challenges persist. Hence, most renal laboratories continue to adopt renal biopsy staining for IHC PLA2R to identify and monitor the disease progression.

## Introduction

Membranous nephropathy (MN) is the second most common cause of nephrotic syndrome after focal segmental glomerulosclerosis in the adult population [1]. Its peak incidence is seen around the age of 40 years in adults, whereas the median age ranges from 50 to 60 years. On average, a mean incidence of eight to 10 cases per one million people is seen worldwide [2]. MN is an idiopathic autoimmune glomerular disease that commonly presents as nephrotic syndrome.

The pathogenesis of MN is the deposition of immune complexes, especially IgG and complement proteins, in the subepithelial region of the glomerular basement membrane, which causes a membrane-like thickening with the formation of spikes [3]. As the immune complex deposition occurs in the subepithelial region, there is no evidence of inflammatory cells in the glomerulus or mesangial region in most cases unless related to other secondary causes. Despite a similar histopathological pattern, MN is an etiologically diverse disease that occurs either in the absence of an established trigger (80% of cases) or in relation to other clinical conditions (20% of cases), such as infections (hepatitis B), lupus erythematosus, cancer, or drugs, thus defining the so-called primary and secondary MN [1,3].

Over the last two decades, researchers have discovered various target antigens using proteomics techniques such as laser microdissection of glomeruli and mass spectrometry of solubilized digested proteins, which have aided in the diagnostic diversity of MN. This has increased the emphasis on detecting target antigens by molecular techniques, serology for specific antibodies, and ancillary studies in renal biopsy [4].

About 14 target antigens have been identified so far, of which phospholipase A2 receptor (PLA2R), thrombospondin type 1 domain containing 7A (THS2DA), NEL-like protein 1 (NELL1), exostosin-1 (EXT1), and exostosin-2 (EXT2) were frequently tested for their association with MN [4,5]. Among these, the two most common antigens are M-type PLA2R and THS2DA, constituting about 70% and 5%, respectively. The discovery of PLA2R antigen dates back to the late 1950s when the Heymann proximal tubule brush border antigen was first identified in rat models. Future studies then led to the identification and isolation of a 185 kDa molecule in 2009, which was subsequently named PLA2R [6].

## Materials and methods

A retrospective observational study was conducted in the Department of Pathology, Sri Ramachandra Medical College and Research Institute (SRIHER), Chennai, India, for a study period of three years from January 2021 to December 2023. All renal biopsy specimens were analyzed, and patients diagnosed with MN were included, while other alternate diagnoses were excluded. Ethical clearance was obtained from the Institutional Ethics Committee (Reference number: CSP-MED/24/AUG/107/242).

A convenience sampling technique was chosen for data collection. The patients' data on sociodemographic status, signs, and symptoms, along with the ancillary studies, were collected from the hospital records and the Laboratory Information System (biochemical and serological values). Renal biopsies were processed and examined under light microscopy. IHC for PLA2R was performed in conformance with the institutes' standard operating procedure using the BSB-129 clone (Bio SB Inc., Goleta, California). Following this, the histopathological slides were retrieved and were analyzed and correlated with the histopathological reports.

Statistical analysis

The collected data were entered in Excel 2018 (Microsoft, Redmond, Washington) and analyzed using SPSS Statistics software version 16 (IBM Inc., Armonk, New York). The Shapiro-Wilk test was used to find the normal distribution. Continuous variables followed a normal distribution, such as age, and were expressed as mean and standard deviation. The description of categorical variables was expressed as frequency and proportion. An independent sample t-test was used to compare two means. The Chi-squared test and Fisher's exact test were employed to compare the distribution of qualitative variables between the groups. All tests were two-tailed, and results were considered statistically significant if the p-value was < 0.05 at a 95% confidence interval.

## Results

In our study, the mean age distribution was 42.85 ± 12.37 years, and the majority of patients were women 36 (55.4%) and men 29 (44.6%). Among 65 cases of MN, 31 (47.7 %) had no detectable underlying cause and were classified as primary MN, while the remaining 34 cases (52.3%) were found to have a coexisting comorbidity and were classified as secondary MN. Upon enumerating these comorbidities, it was found that autoimmune thyroiditis, ovarian carcinoma, and hepatitis B comprised one case (1.5%) each. Eight cases (12.4%) were found to have a history of alternative medicine or non-steroidal anti-inflammatory drugs (NSAID) usage, 12 (18.5%) had systemic lupus erythematosus (SLE), and 11 (16.9%) had type 2 diabetes mellitus and systemic hypertension. In all MN cases, the common clinical presentation observed was facial puffiness and pedal edema, accounting for 59 (90.8%), followed by only pedal edema in five (7.7%) cases and one case (1.5%) with only facial puffiness. In addition, all 65 cases had nephrotic range proteinuria. Among them, serum urea and creatinine levels were analyzed, which showed the median serum urea level as 9 (IQR: 4) mg/dl and the median serum creatinine level as 0.8 (IQR: 0.4) mg/dl. The demographic distribution and basic characteristics are enumerated in Table 1.

**Table 1:** The demographic distribution and basic characteristics among the study participants Continuous variables are expressed as mean and standard deviation; categorical variables in data expressed as frequency (n) and percentage (%) MN - membranous nephropathy; SLE - systemic lupus erythematosus; NSAIDs - non-steroidal anti-inflammatory drugs; DM - diabetes mellitus; SHT -subclinical hypothyroidism

Basic characteristics		Number (n)	Percentage (%)
Mean age in years ± standard deviation		42.85 ± 12.37	
Gender			
Female		36	55.4%
Male		29	44.6%
Subclassification of MN cases			
Primary membranous nephropathy		31	47.7%
Secondary membranous nephropathy	Autoimmune thyroiditis	1	1.5%
	Carcinoma ovary	1	1.5%
	Hepatitis B	1	1.5 %
	Alternative medicine + NSAIDs	8	12.3 %
	SLE	12	18.5 %
	Type 2 DM / SHT	11	16.9 %
Complaints			
Facial puffiness		1	1.5 %
Facial puffiness/ pedal edema		59	90.8 %
Pedal edema		5	7.7 %
Proteinuria	3+	40	61.5 %
	4+	25	38.5 %

In our study, we analyzed the serum levels of antinuclear antibody (ANA), ds DNA, C3, and C4 in 65 cases of MN. Among 31 cases of primary MN, 25 (80.6%) were positive for serum ANA, whereas six (19.4%) were negative. Among 34 cases of secondary MN, 20 (58.8%) were positive, whereas 14 (41.2%) were negative. The differences in ANA positivity among primary and secondary MN was not statistically significant. Serum dsDNA positivity was noted in 12 (35.3%) out of 34 cases of secondary MN, in which all cases were SLE and statistically significant with a p-value of 0.001.

In view of serum C3 and C4 levels, among the 31 primary MN cases, 16 (51.6%) were reported high, one case (3.2%) was reported low, and 14 (45.2%) were reported normal. Among the 34 cases of secondary MN, 16 (47.1%) were reported high in serum C3 and C4, five cases (14.7%) were reported low, and 13 (38.2%) were reported normal. The extended laboratory investigation panel is listed in Table 2.

**Table 2:** Extended laboratory investigation Categorical variables in data expressed as frequency (n) S.ANA - serum antinuclear antibody; S.dsDNA - serum double-stranded DNA; C3, C4 - complements 3 and 4; NSAIDs - non-steroidal anti-inflammatory drugs; SLE - systemic lupus erythematosus; DM - diabetes mellitus; SHT - subclinical hypothyroidism

Basic characteristics		Total number of cases	S.ANA positive	S. ds DNA positive	S.C3/C4		
					High	Low	Normal
Secondary MN	Autoimmune thyroiditis	1	1	0	1	0	0
	Carcinoma ovary	1	0	0	0	0	1
	Hepatitis B	1	0	0	0	0	1
	Alternative medicine + NSAIDs	8	3	0	3	0	5
	SLE	12	12	12	6	5	1
	Type 2 DM / SHT	11	4	0	6	0	5
Sum of all secondary membranous nephropathy cases		34	20	12	16	5	13
Primary membranous nephropathy		31	25	0	16	1	14
Grand total		65	45	12	32	6	27
p-value		-	0.057	0.001	0.277		

**Figure 1:**
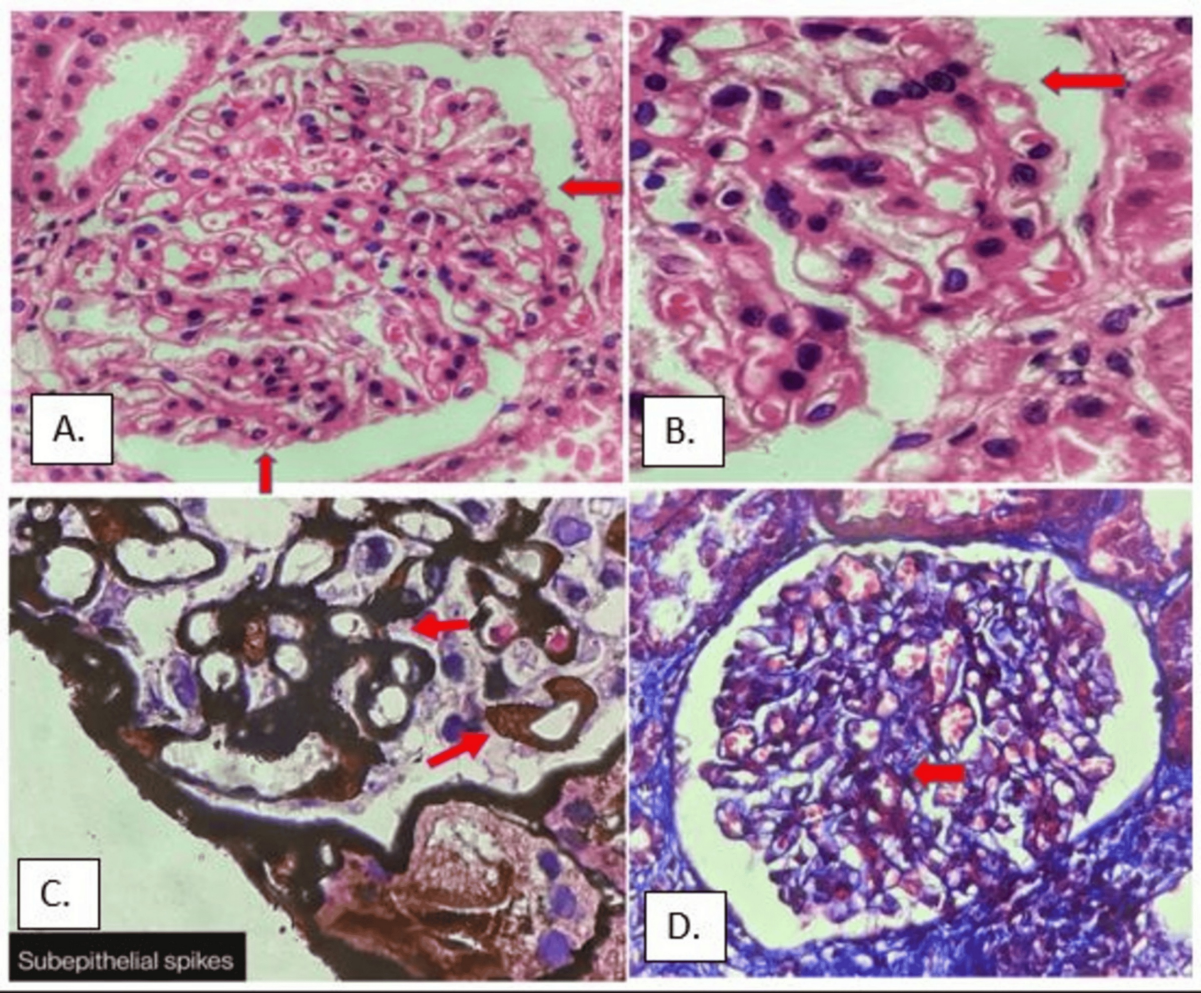
A) H&E showing subepithelial spikes (red arrow) of GBM (100x magnification); B) H&E showing basement membrane thickening and subepithelial spikes (red arrow; 400x magnification); C) JMS stain highlighting subepithelial spikes (red arrow) of GBM (400x magnification); D) MT stain highlighting GBM thickening (red arrow; 400x magnification) GBM - glioblastoma multiforme; JMS - Jones methenamine silver; MT - Masson's trichrome

In our study, renal biopsy specimens of all 65 cases of MN histologically revealed the presence of glomerular basement membrane thickening (GBM). Special stains highlighted the GBM thickening with subepithelial spikes in the Jones methenamine silver (JMS) stain. Thickening of the glomerular basement membrane was highlighted by the Periodic acid-Schiff staining procedure and Masson's trichrome (MT) stain (Figure 1).

The Interstitial Fibrosis and Tubular Atrophy (IFTA) score was analyzed in all 65 cases of MN and scored according to the IFTA scoring system, using an adaptation from the Banff classification [7,8]. Among the 31 primary MN cases, 15 cases (48.4%) had no features of chronicity, five cases (16.1%) had an IFTA score of 0, seven (22.5%) cases had an IFTA score of 1, and four cases (13%) had an IFTA score of 2.

Among the 34 secondary MN cases, 20 cases (58.8%) had no features of chronicity, followed by seven cases (20.6%) with an IFTA score of 0, four cases (11.8%) with an IFTA score of 1, two (5.9%) with an IFTA score of 2, and one case (2.9%) with an IFTA score of 3. Features of chronicity represented as IFTA score were enumerated in Table 3.

**Table 3:** Features of chronicity represented by IFTA score among the study participants Categorical variables in the data expressed as frequency (n) of the total cases IFTA - Interstitial Fibrosis and Tubular Atrophy; MN - membranous nephropathy; NSAIDs - non-steroidal anti-inflammatory drugs; SLE - systemic lupus erythematosus; DM - diabetes mellitus; SHT - subclinical hypothyroidism

Membranous nephropathy		IFTA < 1% (not scored)	IFTA 1 - 10% (score 0)	IFTA 10 -25% (score 1)	IFTA 26 -50% (score 2)	IFTA > 50% (score 3)
Primary MN		15	5	7	4	-
Secondary MN	Autoimmune thyroiditis	1	-	-	-	-
	Ca. ovary	1	-	-	-	-
	Hepatitis B	-	-	1	-	-
	Alternative medicine + NSAIDs	4	2	1	1	-
	SLE	8	1	2	-	1
	T2 DM/ SHT	6	4	-	1	-
Total (n=65)		35	12	11	6	1

**Figure 2:**
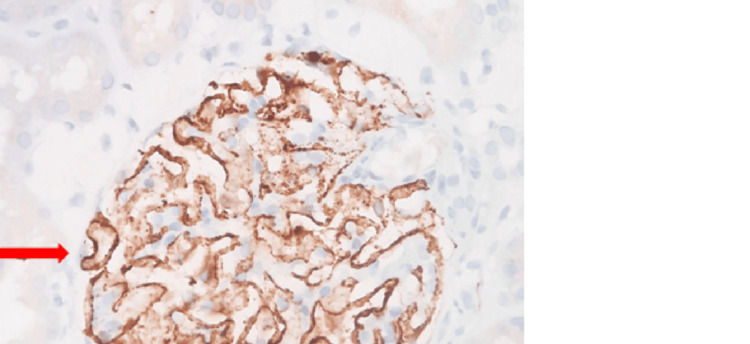
IHC for PLA2R showing diffuse granular positivity (red arrow) of the GBM (400X magnification) IHC - immunohistochemistry; PLA2R - phospholipase A2 receptor; GBM - glioblastoma multiforme

Immunohistochemistry for PLA2R was done in all 65 cases of MN, of which 36 cases (55.4%) were positive and 29 cases (44.6%) were negative. Among the 36 cases (55.4%) of PLA2R positive MN, 26 cases (72.2%) were primary MN, and 10 (27.8%) cases were secondary MN, of which four (40%) were cases of type 2 diabetes mellitus and hypertension, and six (60%) cases had an intake of alternative medications or NSAIDs. Of the 29 (44.6%) of PLA2R negative MN cases, five cases (17.2%) were primary MN, and 24 cases (82.8%) were secondary MN. PLA2R positivity was seen predominantly in primary MN as compared to secondary MN and was statistically significant with a p-value <0.001. PLA2R positive staining pattern of GBM by IHC is depicted in Figure 2. The distribution of PLA2R IHC among the study participants is enumerated in Table 4.

**Table 4:** Distribution of PLA2R staining status by IHC among membranous nephropathy cases Categorical variables expressed in data as frequency (n) and percentage (%) of the total cases MN - membranous nephropathy; IHC - immunohistochemistry; PLA2R - phospholipase A2 receptor

PLA2R status among MN types	Positive	Negative	Total	p-value
Primary MN	26 (72.2%)	5 (17.2%)	31	< 0.001
Secondary MN	10 (27.8%)	24 (82.8%)	34	
Total	36	29	65	

Immunofluorescence (IF) status was analyzed for primary and secondary MN cases. Out of 31 cases of primary MN, 29 cases (93.5%) were positive for C3, and two cases (6.5%) were negative. Among 34 cases of secondary MN, 33 cases (97.1%) were positive for C3, and one (2.9%) case was negative.

**Figure 3:**
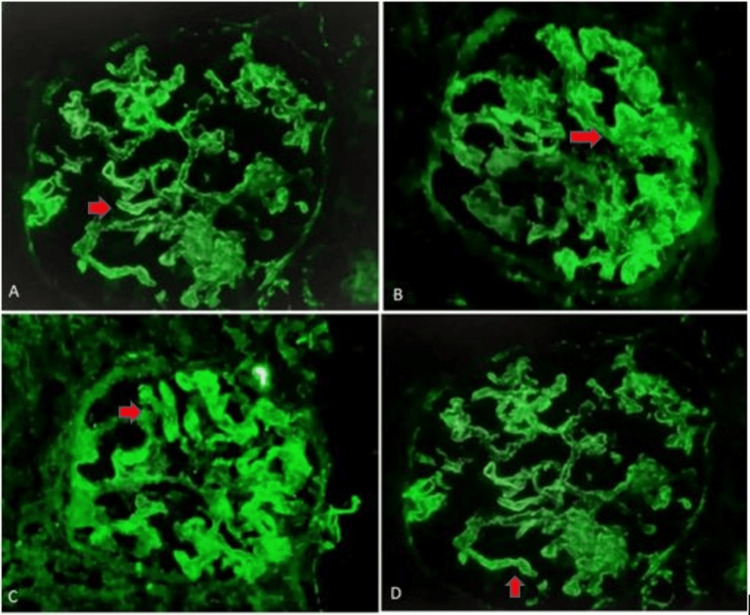
Image A,B,C & D: Represents the diffuse granular staining (Red arrow) of GBM by IF Ig G, C3, Kappa, and Lambda (400X magnification) GBM - glioblastoma multiforme; IgG - immunoglobulin G; C3 - complement 3

IF for C1q, IgG, IgM, kappa, and lambda positivity was seen in all 12 cases of SLE in secondary MN and was statistically significant with a p-value of <0.05. IgG positivity was seen in all cases of primary MN. In secondary MN, 33 cases (97.1%) showed IgG positivity and one case (2.9%) was negative. In one case of autoimmune thyroiditis in secondary MN, IgG with IgA positivity was observed. Kappa and lambda were seen in all 31 cases of primary MN and in 30 (88.2%) of the 34 cases of secondary MN. This difference is statistically significant with a p-value of <0.05. The immunofluorescence status of these cases is enumerated in Table 4. IF staining patterns by various conjugates are represented in Figure 3.

**Table 5:** Immunofluorescence status among membranous nephropathy Categorical variables in data expressed as frequency (n) MN - membranous nephropathy; NSAIDs - non-steroidal anti-inflammatory drugs; SLE - systemic lupus erythematosus; DM - diabetes mellitus; SHT - subclinical hypothyroidism

Basic characteristics		IgG	IgM	IgA	C1q	C3	Kappa	Lambda	Total
Secondary MN	Autoimmune thyroiditis	1	0	1	0	1	0	0	1
	Carcinoma ovary	1	0	0	0	1	0	0	1
	Hepatitis B	1	0	0	0	1	1	1	1
	Alternative medicine + NSAIDs	8	0	0	0	8	7	7	8
	SLE	12	12	0	12	12	12	12	12
	Type 2 DM / SHT	10	0	0	0	10	10	10	11
Sum of all secondary membranous nephropathy cases		33	12	1	12	33	30	30	34
Primary membranous nephropathy		31	0	0	0	29	31	31	31
Grand total		64	12	1	12	62	61	61	65
p-value		0.999	0.002	0.334	<0.001	0.500	0.049	0.049	-

## Discussion

In this study, the mean age of distribution among 65 cases of MN was 43 years, with female predominance. This is in concordance with the study done by Keri et al., which states that the age of presentation of MN is above 40 years [2]. Generally, MN is further subcategorized into primary and secondary MN based on the presence and absence of underlying comorbidities [3-5]. In this study, 31 cases were diagnosed as primary MN and 34 cases as secondary MN. Among the secondary MN cases, the most common underlying etiology was SLE, followed by type 2 diabetes mellitus, hypertension, and alternative medication/ NSAID overuse.

The number of primary and secondary cases of MN in our study is almost proportional except for three cases in secondary. Most of the studies state that primary MN is more predominant than secondary MN. Our study data indicate that the number of cases that presented with nephrotic range proteinuria with underlying chronic disease and drug overuse was more, which accords with the study done by Ronco et al. [3]. This signifies that the incidence of patients who consume traditional medicine is high as compared to Western countries.

In our study, the clinical presentation of all 65 cases of MN showed a history of facial puffiness and pedal edema with nephrotic range proteinuria (>3g/day). They were clinically diagnosed with nephrotic syndrome. In addition, most of the cases (92%) of MN in our study had normal serum urea and creatinine with only slight elevation noted in four (8%) of the cases, in which one case was primary MN and the rest were secondary MN. This shows that not all patients presented with end-stage renal disease and exemplifies the benign nature of the presentation of this disease.

In our study, the biochemical parameters analyzed include serum antinuclear antibody (S.ANA), serum double-stranded DNA (S.dsDNA), and C3 and C4 levels. Serum ANA levels were identified in most of the primary MN. Serum C3 and C4 levels were variable in all cases of MN, with serum C3 levels being high in about 51.6% of cases of primary MN and 47.1% in secondary MN. The high positivity of S.ANA among primary MN cases is consistent with the underlying autoimmune basis of the disease, which reinforces the immune reaction and activation of a complement pathway, as stated by Murtas et al. and many more studies. Histopathological examination of renal biopsies of all these patients showed glomerular basement membrane thickening with subepithelial spikes highlighted by histochemical stains, which are consistent with a diagnosis of MN [6].

In our study, IFTA was analyzed among 65 cases of MN, which showed variable distribution among primary and secondary MN. We scored the IFTA using the Banff scoring system as the basis [7]. Predominant cases in both primary (48.4%) and secondary MN (58.8%) showed no features of chronicity. This signifies the early detection of the disease by renal biopsies and as a prognostic factor in monitoring disease progression [8].

Immunofluorescence of IgG, IgA, IgM, C1q, C3, kappa, and lambda was performed for all 65 cases of MN. Among these immunoglobins and light chains, positive IF was noted for IgG in all cases of primary MN and 97% in secondary MN. In addition, full-house positivity was seen in all cases of SLE-associated secondary MN. Overall, about 80-90% of MN cases showed positivity for C3, IgG, kappa, and lambda, which strongly implies the basis for complement activation and immune system stimulation. This inference is in accordance with the study done by Murtas et al. [9,10].

To test the association of PLA2R with MN, various modalities are available, namely, indirect immunofluorescence with a specificity of 83%, anti-PLA2R Ab detection by enzyme-linked immunoassay (ELISA; 98% specificity), and immunohistochemistry in renal biopsies with a specificity of 96.6% [11,12]. Although there are various modes for testing the association of PLA2R with MN, certain limitations exist in following these modalities due to cost-effectiveness and accessibility in all laboratories. In addition, certain diagnostic challenges exist in interpreting the anti-PLA2R antibodies (anti-PLA2R Ab), which include the absence of serum anti-PLA2R Ab, suggests that the patient may be in the remission phase, has early-stage disease, or has been treated with immunosuppressants or even lapsed between the renal biopsy and antibody estimation [10,11,12]. Thus, IHC staining for PLA2R is consistently followed due to certain inconclusive results obtained from other modes because of the heterogeneity of the patient's clinical presentation. 

In this study we analyzed the expression of PLA2R by immunohistochemistry in 65 cases of MN. Of these, 55.4% showed positivity for PLA2R, of which 72.2% were positive among primary MN and 27.7% were secondary MN, which is statistically significant with a p-value <0.001. The results are in accordance with Roy et al. [12,13]. The remaining cases of MN showed negative PLA2R staining, which warrants analysis of other target antigens (THS2DA, NELL1, EXT1, EXT2, PCDH7) that could instead be the underlying antigen causing MN in such cases [14,15]. In a study published by Beck et al., clinical trials are still being performed to implement serum anti-PLA2R Ab as a sole marker for classifying and staging MN. Hence, it is always better to follow an algorithmic approach in utilizing modern techniques for disease stratification. Therefore, most renal laboratories continue to follow renal biopsy staining for PLA2R by IHC to monitor the disease's behavior and progression.

In our study, we adopted the traditional method to broadly classify MN based on the clinical history for better comprehension. However, recent advancements in modern techniques have led to the invention of new target antigens, which directed to a more pathogenetically specific reclassification of MN. The importance of this novel classification based on the target antigen helps us with potential implications for targeted therapies. With regards to primary MN, immunosuppressant rituximab, which mainly acts against B cells, is currently employed. In secondary MN, the underlying etiology has to be addressed and treated [16]. 

## Conclusions

Membranous nephropathy is considered a pattern of glomerular injury rather than a disease. Therefore, it is essential to understand the genesis of this disease and carefully identify the underlying factors for the subsequent development of renal impairment that progresses to end-stage renal disease if not diagnosed early. Recent studies have insisted on reclassifying MN more precisely due to the discovery of various target antigens. Each patient has unique clinical and prognostic factors, which are found to be influenced by the various target antigens. This study revealed that primary MN is more frequently associated with the positive immunohistochemical expression of PLA2R with statistical significance (p-value <0.001). This helps us in subcategorizing MN into antigen-specific (PLA2R positive and negative cases) and allows us ongoing validation of PLA2R IHC as a diagnostic marker, which aids in monitoring the disease progression, remission, and recurrence. In addition, to relate the association of other novel antigens with MN, more trials and studies must be encouraged with large study groups to prove the association of various target antigens by multiple step-based algorithms to provide the utmost benefits for patients’ clinical outcomes.
